# Identification of diverse antibiotic resistant bacteria in agricultural soil with H_2_^18^O stable isotope probing combined with high-throughput sequencing

**DOI:** 10.1186/s40793-023-00489-7

**Published:** 2023-04-18

**Authors:** Marcela Hernández, Shamik Roy, C. William Keevil, Marc G. Dumont

**Affiliations:** 1 School of Biological Sciences, University of Southampton, Southampton, SO17 1BJ UK; 2 School of Biological Sciences, University of East Anglia, Norwich, NR4 7TJ UK

**Keywords:** Antimicrobial resistant bacteria, Soil, Antibiotics, Pathogens, DNA stable isotope probing, High-throughput sequencing, Metagenomics

## Abstract

**Background:**

We aimed to identify bacteria able to grow in the presence of several antibiotics including the ultra-broad-spectrum antibiotic meropenem in a British agricultural soil by combining DNA stable isotope probing (SIP) with high throughput sequencing. Soil was incubated with cefotaxime, meropenem, ciprofloxacin and trimethoprim in ^18^O-water. Metagenomes and the V4 region of the 16S rRNA gene from the labelled “heavy” and the unlabelled “light” SIP fractions were sequenced.

**Results:**

An increase of the 16S rRNA copy numbers in the “heavy” fractions of the treatments with ^18^O-water compared with their controls was detected. The treatments resulted in differences in the community composition of bacteria. Members of the phyla Acidobacteriota (formally Acidobacteria) were highly abundant after two days of incubation with antibiotics. Pseudomonadota (formally Proteobacteria) including *Stenotrophomonas* were prominent after four days of incubation. Furthermore, a metagenome-assembled genome (MAG-1) from the genus *Stenotrophomonas* (90.7% complete) was retrieved from the heavy fraction. Finally, 11 antimicrobial resistance genes (ARGs) were identified in the unbinned-assembled heavy fractions, and 10 ARGs were identified in MAG-1. In comparison, only two ARGs from the unbinned-assembled light fractions were identified.

**Conclusions:**

The results indicate that both non-pathogenic soil-dwelling bacteria as well as potential clinical pathogens are present in this agricultural soil and several ARGs were identified from the labelled communities, but it is still unclear if horizontal gene transfer between these groups can occur.

**Supplementary Information:**

The online version contains supplementary material available at 10.1186/s40793-023-00489-7.

## Introduction

Soil represents a reservoir of antimicrobial resistance genes (ARG) that may in part have originated as a defence mechanism against antimicrobial products secreted by competing microbes. In addition, the release of antibiotics from clinical and veterinary use may also be driving antimicrobial resistance (AMR) and spread within terrestrial ecosystems [1]. Therefore, ARGs can be detected in all soils including garden soil [2], agricultural soil [3], forest soil [4], grasslands [5], and Antarctic soils [6]. There is also a wide diversity of ARGs in soils representing up to 32% of the overall diversity [7]. In addition, a previous study reported the importance of low abundance antimicrobial-resistant microbes in soil-plant systems for the spread of AMR [8].

The potential transmission of AMR back to humans through a soil-microbe-animal-plant nexus endangers public health, since the spread of AMR could push us to the post-antibiotic era. The drivers, or mechanisms, of the spread of AMR in soils challenged with antibiotics remains to be determined. Deciphering this knowledge gap is crucial for us to develop strategies to alleviate the spread of AMR in terrestrial ecosystems.

It has been hypothesised that the spread of AMR in soil is primarily driven by two linked processes that can operate in tandem to alter the soil resistome [1, 9]. One process is horizontal gene transfer (HGT) of antimicrobial resistance genes (ARGs) between microbial community members. The second process is the directional selection of antimicrobial resistant microbes under antibiotic selection. This could be either due to incorporation of microbiomes derived from anthropogenic sources (e.g., organic fertiliser), or selection and proliferation of naturally resistant microbiota. We are now beginning to understand how HGT can facilitate the spread of AMR in pristine environments [6, 10]. For instance, the *bla*_NDM-1_ gene that confers resistance to carbapenem (antibiotic of last resort) is now ubiquitous due to successive and distinct HGT events [11, 12]. On the other hand, there is limited knowledge about the community composition of the microbiome that can resist antibiotic in soil. One of the main reasons could be the large abundance of extracellular DNA (eDNA) in soil, which cannot distinguish active antimicrobial resistant microbes from dead/dormant antimicrobial sensitive microbes [13, 14]. This could be a reason why some studies have reported contradictory results of no change to complete change in microbiomes upon antibiotic addition [5, 15].

Agricultural ecosystems represent 38% of the Earth’s ice-free terrestrial surface — the largest use of land on the planet [16]. Sustainable agricultural practice includes the adoption of organic fertilisers instead of chemical fertilisers as a source of nutrients to maintain or increase crop yield [17]. However, the build-up of antibiotics and ARGs in organic fertilisers, such as livestock manure and sewage sludge, can spread antimicrobial resistance in agricultural soils [1, 9, 13, 18]. Since the endemic resistant microbes in soils are one of the major determinants of AMR spread, it is crucial to identify the active fraction of the soil microbial community that can grow in the presence of antibiotics.

Stable isotope probing (SIP) with [^18^O]-water is a unique approach to identify the active AMR microbes. SIP is a cultivation-independent approach that requires the addition of stable-isotope-enriched substrates (e.g., ^13^C-methane, ^18^O-water) to environmental samples followed by the analyses of labelled DNA or RNA [19]. SIP techniques can target phylogenetically constrained metabolic processes (e.g., ethane oxidation), where from a diverse pool of active microbial community only those microbial guilds that can assimilate and subsequently incorporate the labelled substrate into their biomolecules such as DNA and RNA are identified. In contrast, SIP-H_2_^18^O as a substrate can potentially label all metabolically active or growing microbes since water is a prerequisite for growth and cellular maintenance [20, 21]. Fast-growing microbes are labelled first, but eventually all active microbes are expected to contain isotope-enriched DNA. Additionally, ^18^O has two more neutrons than naturally abundant ^16^O, whereas ^2^H, ^13^C and ^15^N has only one additional neutron compared to their naturally abundant counterparts (^1^H, ^12^C and ^14^N). This can potentially increase the degree of physical separation of labelled ^18^O-DNA from unlabelled DNA during isopycnic centrifugation in SIP. As a result, the SIP-H_2_^18^O has been used as a robust method to identify the active microbes in soil including their response to nutrient addition [22], rewetting [21, 23], and warming [24].

In this study we combined antibiotic selection and SIP-H_2_^18^O to identify active and growing microbial communities in an agricultural soil. Antibiotic selection in the experiment ensured only antimicrobial-resistant microbes grew and simultaneously killed or inhibited the growth of sensitive microbes. We also used agricultural soil with no history of antibiotic addition either directly, or indirectly via organic fertilisers. This was done to reduce the bias in identification that can be introduced from long-term exposure of microbes to exogenous antibiotics as it may already have selected for a resistant microbial community. Our objectives for this study were to investigate whether bacteria in agricultural soil with no-antibiotic history can grow if challenged with antibiotic; secondly, if present, to identify the microbes and the ARGs that confer resistance. We hypothesise that diverse resistant microbes can be identified in soil and their identification will help to understand the potential for AMR spread.

## Results

To evaluate whether microbes in an agricultural soil with no-antibiotic history can grow if challenged with antibiotic, agricultural soils were incubated with an antibiotic cocktail of meropenem (mem), cefotaxime (ctx), ciprofloxacin (cip) and trimethoprim (tmp), along with H_2_^18^O or natural isotope abundance water (referred to as H_2_^16^O). Here we report the results after four days of incubation with antibiotic addition at 0 and 48 h time-points. A total of 18 CsCl gradient fractions were collected following ultracentrifugation and the 16S rRNA gene copy numbers (bacterial abundance) were analysed for each experimental setup.

**Fig. 1:**
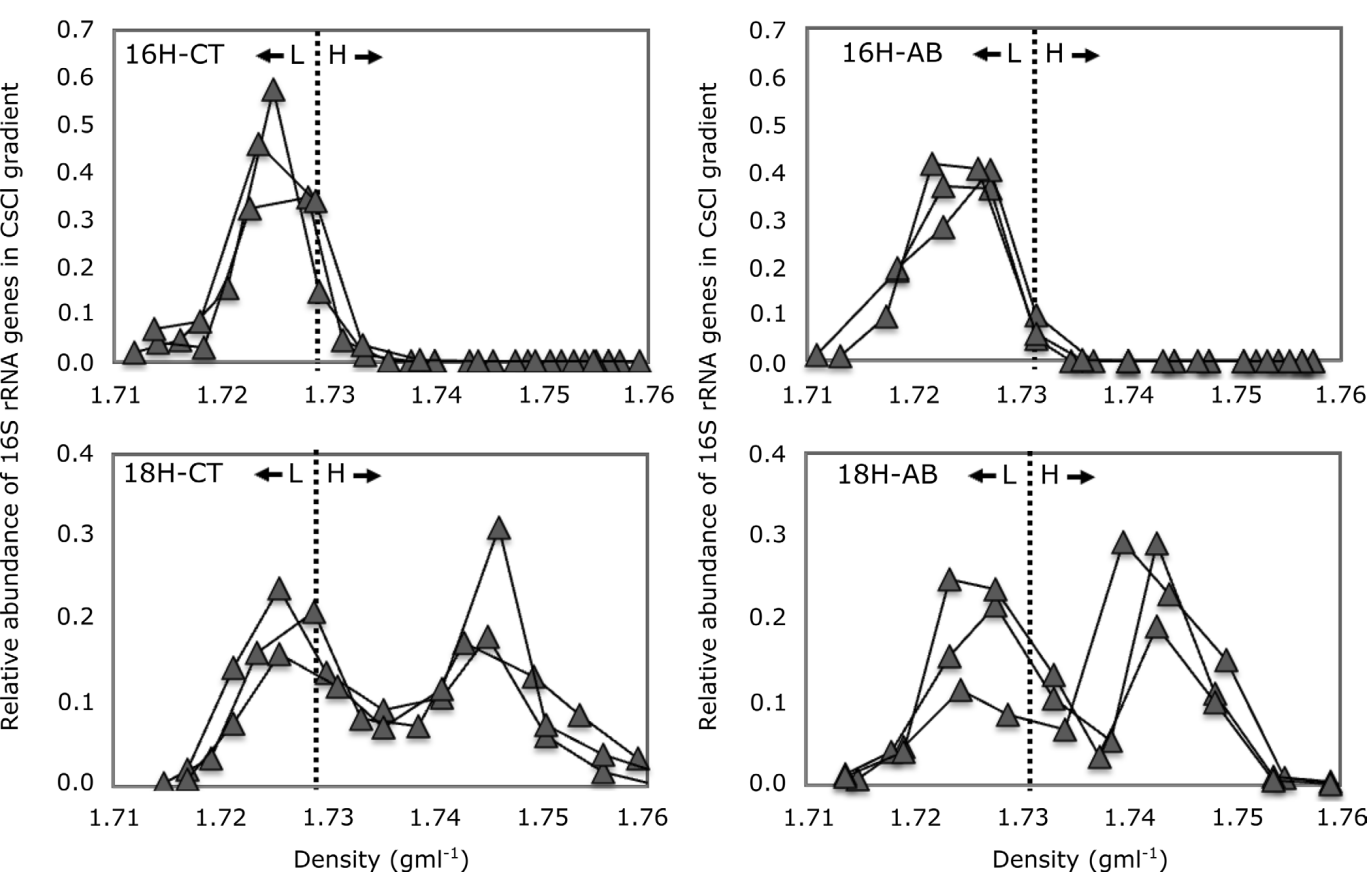
Abundance of bacterial 16S rRNA genes in CsCl density gradients after ^16^O-/^18^O-H_2_O incubation. Vertical dotted lines demarcate the heavy (H) fractions from light (L) fractions. Each line represents a sample. 16 H-CT: soils incubated in the presence of natural isotope abundance water (H_2_^16^O) without antibiotics (control); 16 H-AB: incubation in the presence of H_2_^16^O and antibiotics; 18 H-CT: incubation in the presence of H_2_^18^O without antibiotics (control); 18 H-AB: incubation in the presence of H_2_^18^O and antibiotics

Incubation with H_2_^18^O increased the overall buoyant density of the extracted DNA as compared to the H_2_^16^O controls (Fig. 1). ^18^O-labelled DNA (heavy fractions) resided in fractions with densities 1.73 g ml^-1^ and above, whereas unlabelled DNA (light fractions) resided in fractions with densities 1.729 g ml^-1^ and lower (Fig. 1). This indicates that bacteria were actively incorporating ^18^O into their DNA. Here, the heavy fraction indicates active or growing microbes, whereas the light fraction indicates dormant or dead microbes.

After four days of incubation with H_2_^18^O there was a large abundance of bacterial 16S rRNA gene copies in the heavy fraction as compared to the heavy fraction of samples incubated with H_2_^16^O (Fig. 1). This was the case for both the antibiotic treatment and no-antibiotic controls suggesting there was substantial bacterial growth in the presence of antibiotics.

**Fig. 2:**
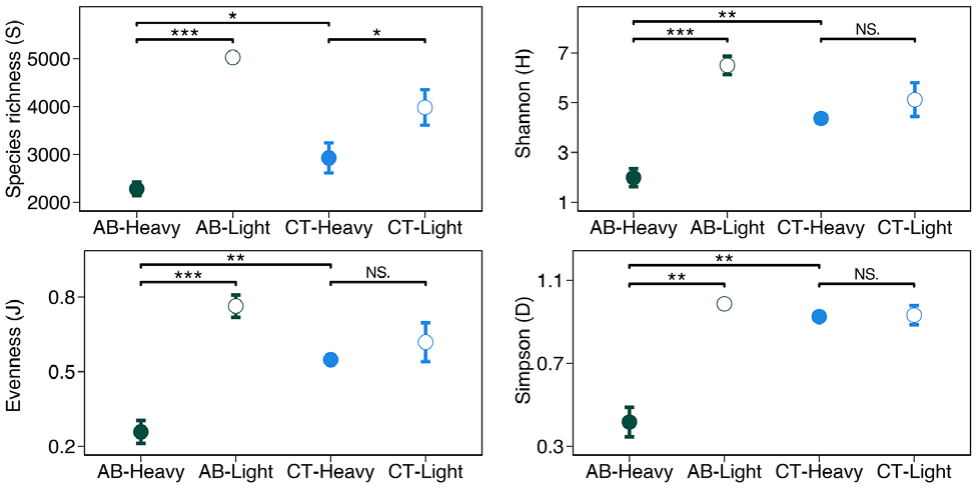
Alpha-diversity of 16S rRNA gene sequences from the “heavy” and “light” fractions of DNA extracted from soils incubated with H_2_^18^O in the presence (AB) or absence (CT) of antibiotics. Alpha-diversity is summarised as species richness (S), Shannon diversity (H), evenness (J), and Simpson (D). “*” corresponds to p-value < 0.05 from pairwise t-test; “**” corresponds to p-value < 0.01; “***” corresponds to p-value < 0.001; “NS.” corresponds to p-value > 0.05

Species richness was significantly lower in the heavy fractions (2278 ± 142, mean ± 95% confidence interval) than light fractions (5025 ± 99) for antibiotics (AB) treated soil (p < 0.001). Similarly for CT treatment (i.e., control: soil without antibiotics), the species richness was significantly lower (p = 0.013) in the heavy fractions (2926 ± 314) than in the light fractions (3980 ± 370). Shannon diversity (*H*) was lower (p < 0.001) in the heavy fractions (1.99 ± 0.36) than light fractions (6.50 ± 0.37) for AB treated soil. However, for CT, Shannon diversity (*H*) did not differ (p = 0.157) between heavy fractions (4.37 ± 0.12) and light fractions (5.12 ± 0.68). Evenness (*J*) indexes were lower (p < 0.001) in the heavy fractions (0.42 ± 0.07) than in light fractions (0.98 ± 0.01) for AB treated soil. Contrarily, for control treatments (CT), evenness (*J*) did not differ (p = 0.219) between heavy fractions (0.93 ± 0.01) and light fractions (0.93 ± 0.05) (Fig. 2).

When comparing the heavy fractions of AB and CT treatments, species richness was lower (p = 0.039) for heavy fractions for AB treatments (2278 ± 142) than CT treatments (2926 ± 314). Similarly, Shannon diversity was lower (p = 0.003) for AB treatments (1.99 ± 0.36) than CT treatments (4.37 ± 0.12). Evenness was also lower (p = 0.005) for AB treatments (0.42 ± 0.07) than CT treatments (0.93 ± 0.01) (Fig. 2). Finally, the coefficient of variation (CV) for all alpha diversity indices across all the treatments ranged from 0.4 to 16.0% (Fig. 2).

**Fig. 3:**
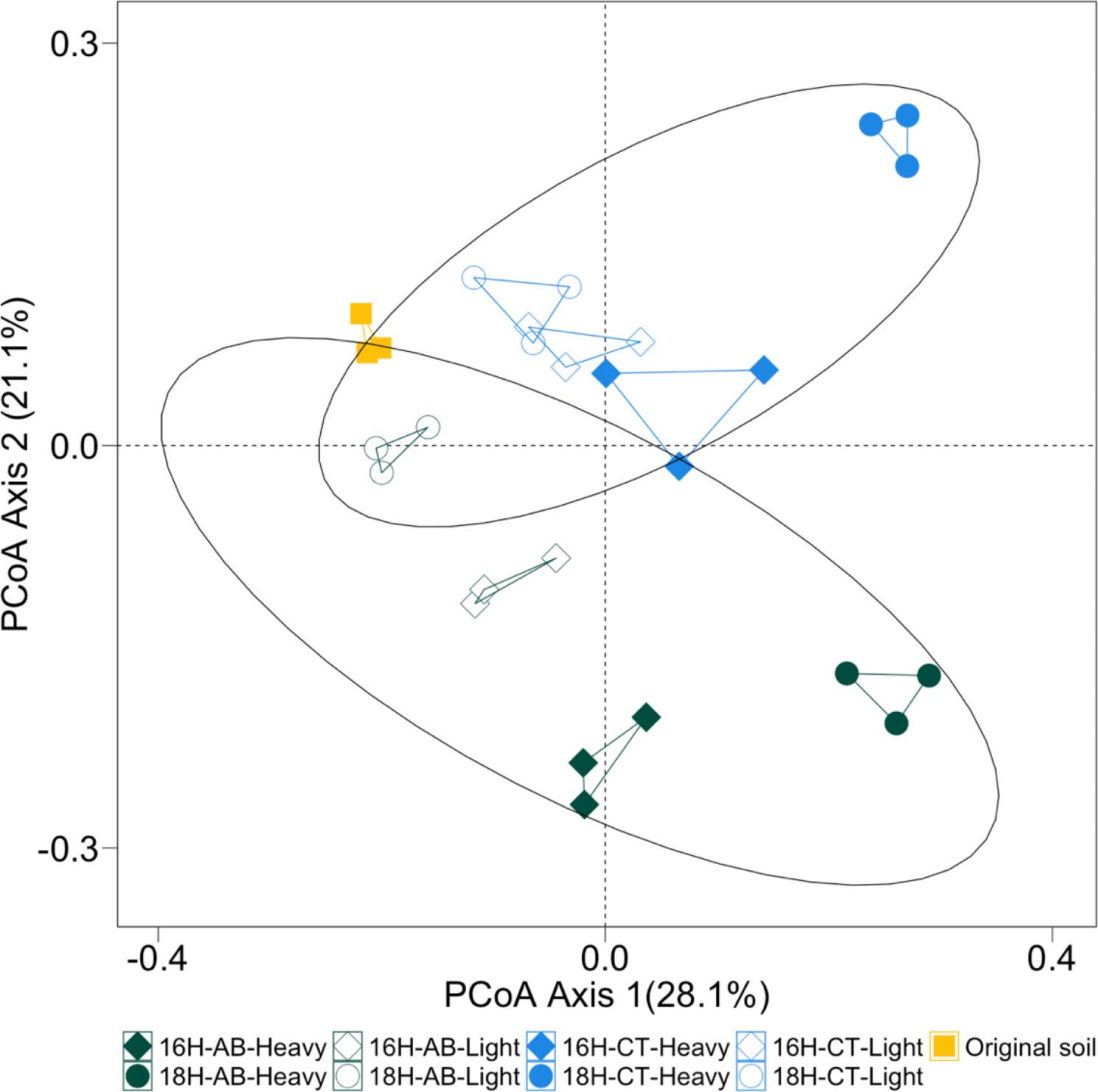
Principal coordinate analysis (PCoA) plots of bacterial OTUs (97% sequence similarity) derived from 16S rRNA genes extracted from soil. The ellipse indicates the difference between microbial communities in the presence or absence of antibiotics in the PCoA space (PERMANOVA: p = 0.001, R^2^ = 0.26). The legend indicates the origin of the samples. 16 H: incubation with H_2_^16^O; 18 H: incubation with H_2_^18^O; AB: incubation with antibiotics; CT: incubation without antibiotics; Heavy: “heavy” fractions of the extracted soil DNA; Light: “light” fractions of the extracted soil DNA.

**Fig. 4:**
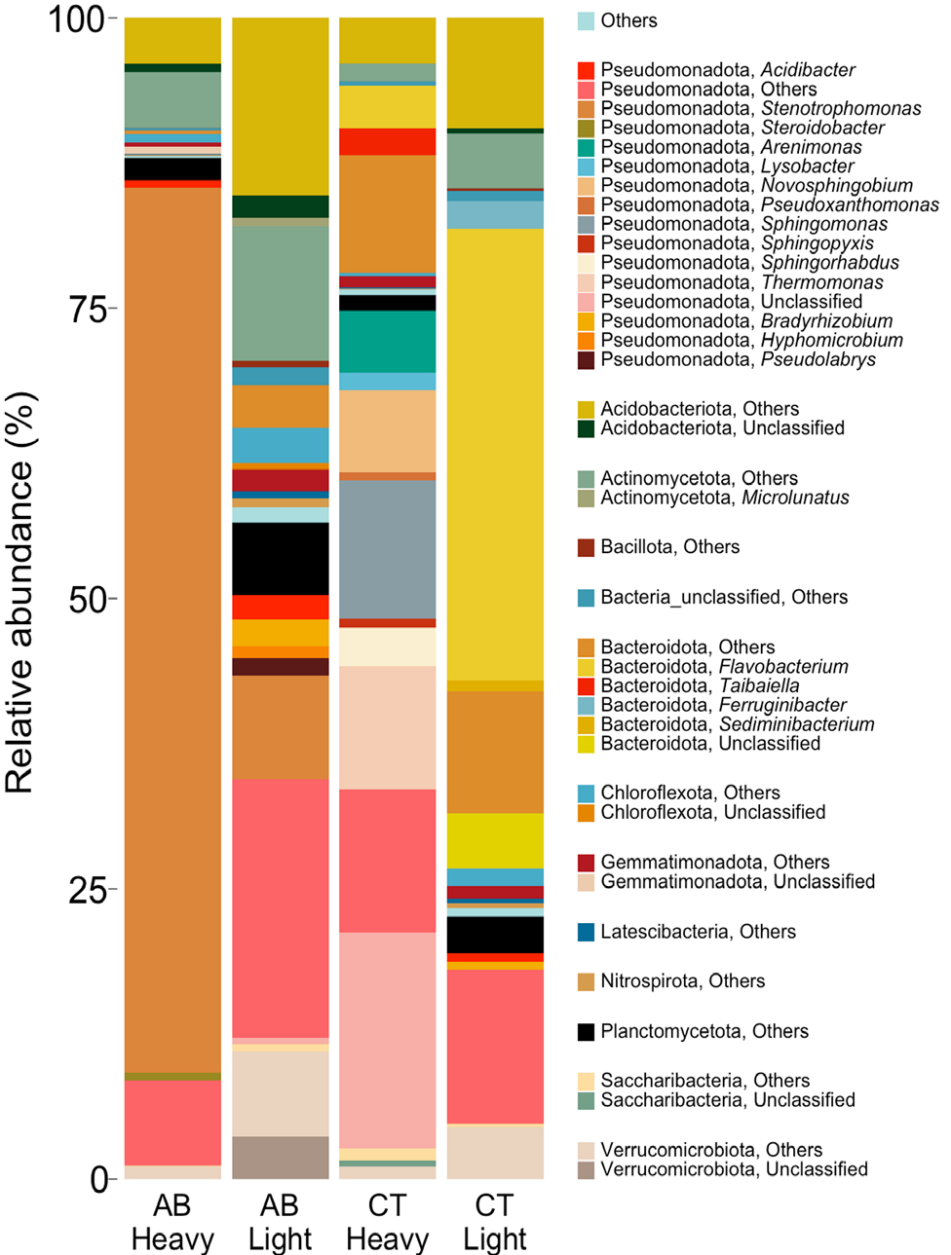
Relative abundance of microbial communities at the phylum level identified in the “heavy” and “light” fractions of DNA extracted from soils incubated with H_2_^18^O in the presence (AB) or absence (CT) of antibiotics. ‘Unclassified’ taxa are those OTUs that were not classified at the genus level. ‘Others’ are those OTUs that were classified but the total abundance was less than 0.5% of all OTUs

The microbial community composition was consistent for all replicates of both heavy and light fractions across all the treatments. The community composition for heavy and light fractions of AB and CT when incubated with H_2_^18^O were different as they clustered separately (Fig. 3). Community composition of the light fraction in H_2_^18^O incubated CT soil (18 H-CT-Light) was similar to both the heavy (16 H-CT-Heavy) and light (16 H-CT-Light) fraction in H_2_^16^O incubated CT soils as shown by their proximity in the PCoA plot and the relative abundance profile (Fig. 4, S1). Together, these three fractions (18 H-CT-Light, 16 H-CT-Heavy, 16 H-CT-Light) along with the light fraction of H_2_^16^O incubated AB soil (16 H-AB-Heavy) were similar to the composition of the original soil.

The community in the heavy fractions of AB incubated with H_2_^18^O were dominated by high relative abundances of Pseudomonadota (84.9 ± 2.86%), Actinomycetota (formally Actinobacteria, 4.8 ± 1.42%), Acidobacteriota (4.7 ± 0.93%), Planctomycetota (formally Planctomycetes, 1.9 ± 0.42%), Verrucomicrobiota (formally Verrucomicrobia, 1.2%), and Gemmatimonadota (formally Gemmatimonadetes, 1.1 ± 0.10%). *Stenotrophomonas* (Pseudomonadota, 76 ± 4.67%) was the most abundant genus (Fig. 4). In contrast, the heavy fractions for CT treatments were dominated mainly by Pseudomonadota (72.1 ± 7.9%), Bacteroidota (formally Bacteroidetes, 16.1 ± 5.03%), Acidobacteriota (3.9 ± 0.83%), Saccharibacteria (2.3 ± 2.22%), Actinomycetota (1.5 ± 0.38%), Planctomycetota (1.3 ± 0.15%), Verrucomicrobiota (1.24%), and Gemmatimonadota (1.1%). Here, *unclassified* (Pseudomonadota, 17.18%), *Sphingomonas* (Pseudomonadota, 11.9%), *Thermomonas* (Pseudomonadota, 10.8%), *Arenimonas* (Pseudomonadota, 5.28%), *Novosphingobium* (Pseudomonadota, 7.03%) were the abundant genera (Fig. 4).

The relative abundance in the light fractions of AB were dominated by Pseudomonadota (38.7 ± 5.38%), Acidobacteriota (17.2 ± 0.62%), Actinomycetota (12.3 ± 4.24%), Verrucomicrobiota (10.9 ± 1.01%), Planctomycetota (6.2 ± 1.17%), Bacteroidota (3.7 ± 0.29%), Chloroflexota (formally Chloroflexi, 3.6 ± 0.68%), and Gemmatimonadota (1.9 ± 0.19%). The most abundant genera were *Stenotrophomonas* (Pseudomonadota, 8.9%), *Bradyrhizobium* (Pseudomonadota, 2.3%, only in one replicate), and *Acidibacter* (Pseudomonadota, 2.1%) (Fig. 4). In contrast, the light fractions of CT were dominated by Bacteroidota (57.5 ± 8.54%), Pseudomonadota (14.7 ± 2.46%), Acidobacteriota (9.9 ± 2.59%), Actinomycetota (4.7 ± 1.79%), Verrucomicrobiota (4.4 ± 1.29%), Planctomycetota (3.1 ± 0.62%), Chloroflexota (1.52 ± 0.40%), and Gemmatimonadota (1.1 ± 0.12%). The most abundant genus in this treatment was *Flavobacterium* (Pseudomonadota, 38.91 ± 4.67%) (Fig. 4).

**Fig. 5:**
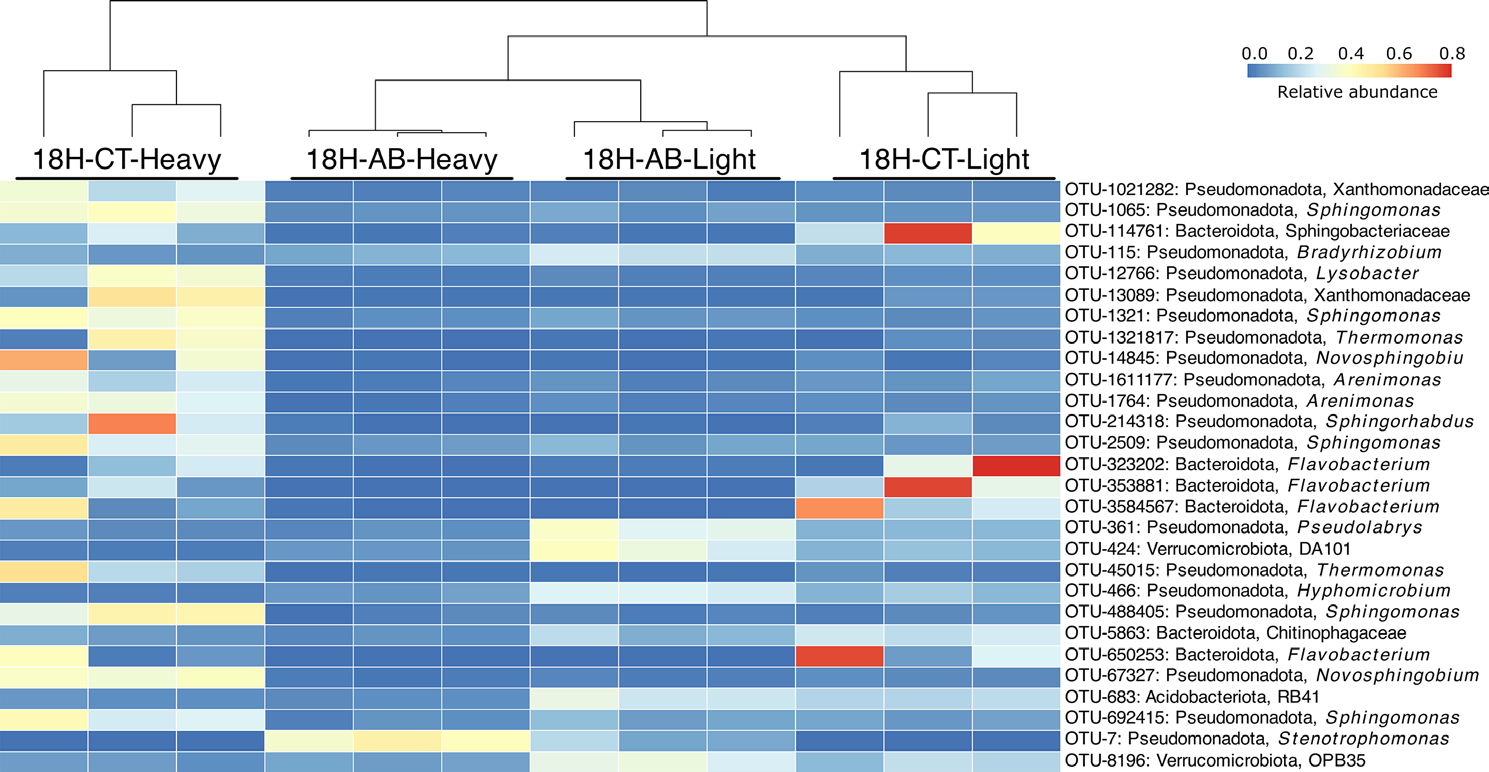
Heatmap of selected bacterial OTUs identified in the “heavy” and “light” fractions of DNA extracted from soils incubated with H_2_^18^O in the presence (AB) or absence (CT) of antibiotics. 18 H-AB-heavy indicates “heavy” fractions treated with antibiotic; 18 H-AB-light indicates “light” fractions treated with antibiotic; 18 H-CT-heavy indicates “heavy” fractions without antibiotic treatment; 18 H-CT-light indicates “light” fractions without antibiotic treatment. The coloured scale represents the relative abundance of the 28 selected OTUs.

A heatmap was created to visualise and compare the abundance of the 20 OTUs that explains the most variation in the axis-1 and axis-2 of the PCA ordination. Out of a total of 28 OTUs selected, 19 OTUs belonged to Pseudomonadota, followed by six OTUs of Bacteroidota, two OTUs of Verrucomicrobiota, and one OTU of Acidobacteriota. *Stenotrophomonas* (Pseudomonadota; OTU-7) was dominant in the heavy fraction of AB compared to heavy fraction of CT. On the other hand, *Sphingomonas* (Pseudomonadota; OTU-1065, OTU-1321, OTU-2509, OTU-488,405, OTU-692,415), *Lysobacter* (Pseudomonadota; OTU-12,766), *Novosphingobium* (Pseudomonadota; OTU-14,845), Xanthomonadaceae (Pseudomonadota; OTU-13,089), *Arenimonas* (Pseudomonadota; OTU-1764) were dominant in heavy fraction of CT compared to AB. Additionally, *Pseudolabrys* (Pseudomonadota; OTU-1764), DA101 (Verrucomicrobiota; OTU-424), OPB35 (Verrucomicrobiota; OTU-8196) were dominant in light fractions of AB compared to heavy fractions of AB (Fig. 5).

**Fig. 6:**
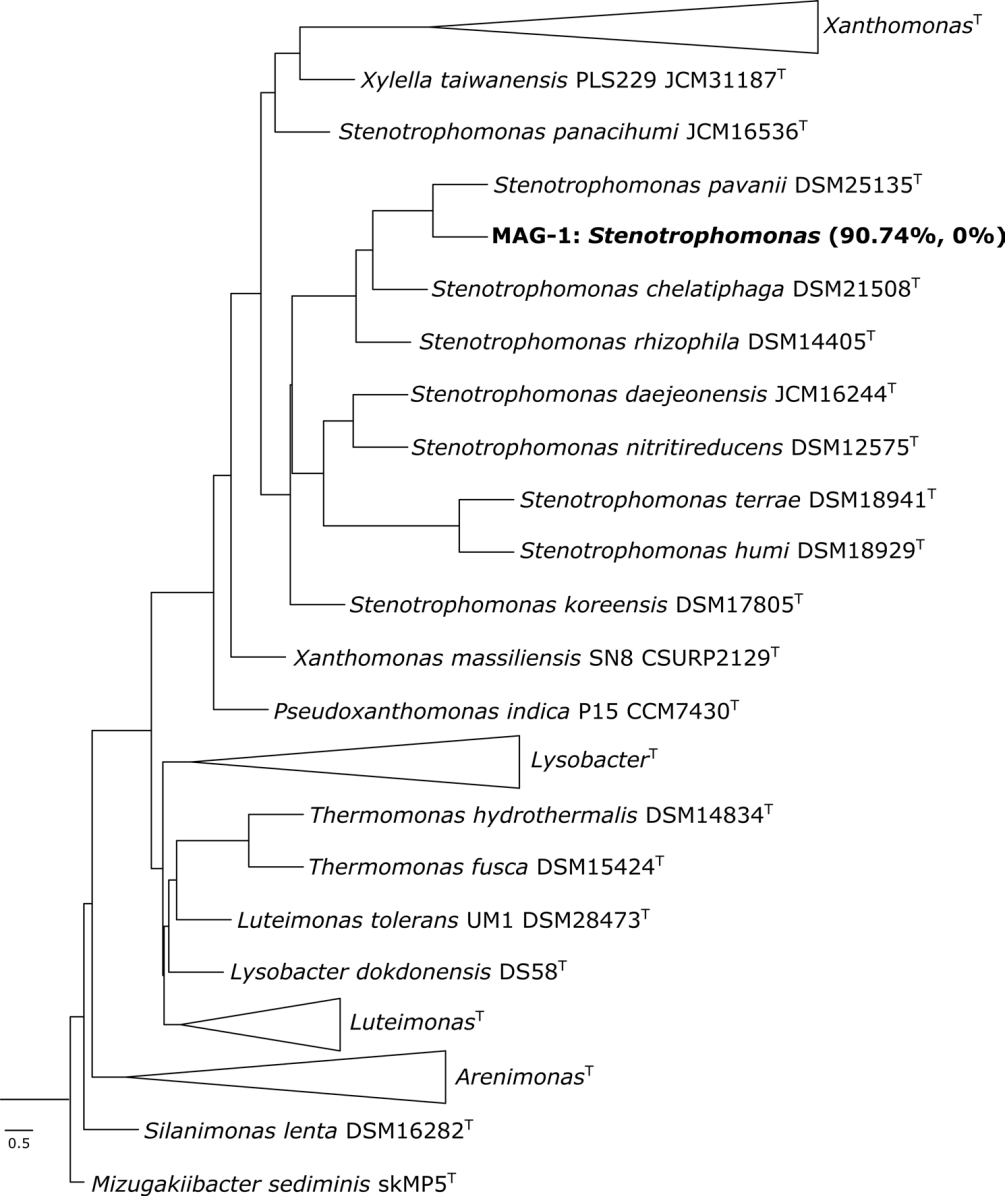
Multi-locus phylogenetic tree of the MAG-1 using autoMLST. 98 conserved housekeeping genes were used for the analyses. MAG-1 is indicated together with its completeness and contamination

The DNA of both heavy and light fractions of soil when incubated with ^18^O-labelled water in the presence of antibiotics were sequenced individually using high-throughput sequencing. After genome binning of both heavy and light fractions, one qualified metagenome-assembled genome (MAG) was generated with 90.7% completeness and 0% contamination. The MAG was affiliated to *Stenotrophomonas* (Pseudomonadota) (Fig. 6). This is in-sync with the results of 16S rRNA gene sequencing that also showed *Stenotrophomonas* (Pseudomonadota) as the dominant genus when incubated with antibiotics (Figs. 4 and 5).

Analysis of the unbinned-assembled genomes of light and heavy fractions, along with the genome of MAG-1 helped understand whether the microbial community exposed to antibiotic contained antimicrobial resistance genes (ARGs) to survive the stress of antibiotics. The presence of *aph(3’)-IIc*, with 85.46% similarity to *Stenotrophomonas maltophilia* strain K279a was observed in the heavy fraction and MAG-1, and no presence of this gene was observed in the light fraction. *aph(3’)-IIc* encodes the aminoglycoside phosphotransferase enzyme that confers resistance to antibiotics in the aminoglycoside class (butirosin, paromycin, kanamycin, neomycin) among others. Similarly, the presence of *oqxB*, with 76.28% similarity to *Escherichia coli* plasmid pOLA52 in heavy fraction and MAG-1, which was also absent in the light fraction was also observed. The *oqxB* gene encodes an efflux pump that confers resistance to amphenicol class antibiotics (e.g., chloramphenicol), disinfectants (e.g., benzalkonium chloride, cetylpyridinium chloride), quinolone class antibiotics (e.g., ciprofloxacin, nalidixic acid), trimethoprim, and others. However, *dfrB3*, which encodes dihydrofolate reductase that confers resistance to trimethoprim, was found with a 90.14% similarity with the plasmid R751 in *Klebsiella aerogenes* only in the light fraction. The presence of ARGs that confer resistance to beta-lactam were also found in both the light and heavy fractions, but not in MAG-1. For example, *blaTEM-181* in the light fraction was found with a 99.86% similarity with vector pUC-3GLA, and *blaL1* in the heavy fraction with 85.84% of similarity with a beta-lactamase gene in *Stenotrophomonas maltophilia* strain K1. No ARG conferring beta-lactam resistance was present in MAG-1 (Table 1).

**Table 1:** Antimicrobial resistance genes found in the unbinned-assembled reads (from heavy and light fractions) and MAG-1

Antimicrobial	Class	light	heavy	MAG-1	Gene (Similarity (%) / Reference*)		
					Light	Heavy	MAG-1
Butirosin	Aminoglycoside		X	X		aph(3’)-IIc (85.46% / AM743169)	aph(3’)-IIc (85.46% / AM743169)
Paromomycin			X	X			
Kanamycin			X	X			
Neomycin			X	X			
Chloramphenicol	Amphenicol		X	X		OqxB (76.28% / EU370913)	OqxB (76.28% / EU370913)
Benzylkonium chloride	Quaternary ammonium compound		X	X			
Cetylpyridinium chloride	Quaternary ammonium compound		X	X			
Ciprofloxacin	Quinolone		X	X			
Nalidixic acid	Quinolone		X	X			
Trimethoprim	Folate pathway antagonist	X	X	X	dfrB3 (90.14% / X72585)	OqxB (76.28% / EU370913)	OqxB (76.28% / EU370913)
Unknown beta-lactam	Beta-lactam	X	X		blaTEM-181 (99.86% / KM977568)	blaL1 (85.84% / EF126059)	

* AM743169: *Stenotrophomonas maltophilia* strain K279a; EU370913: *Escherichia coli* plasmid pOLA52; X72585: *Klebsiella aerogenes* plasmid R751 genes from integron element; KM977568: Cloning and transformation vector pUC-3GLA; EF126059: *Stenotrophomonas maltophilia* strain K1 (beta-lactamase gene)

## Discussion

In this study we used DNA-SIP with H_2_^18^O to identify the antimicrobial resistant microbes from the active pool of an agricultural soil that was not previously exposed to antibiotics. The results showed that microbes can grow in the presence of antibiotic even in the agricultural soil with no-antibiotic history (Fig. 1). On the other hand, not all active microbes are antimicrobial resistant, since community composition was different between antibiotic treated and untreated soil (Figs. 2, 3, 4 and 5). The metagenomic analyses revealed the presence of ARGs in the active resistant microbial community (Table 1). Additionally, a MAG belonging to *Stenotrophomonas* was found in the heavy fraction after incubation with H_2_^18^O and antibiotics. The study highlights the ability of DNA-SIP with H_2_^18^O to identify active antimicrobial resistant microbes.

The results showed that soils without prior exposure to antibiotic can harbour microbes that can become active and enriched in the presence of antibiotics during a short period of time, in this case after four days of incubation. This suggests that soils contain a resistome of antimicrobial resistance, and the microbiome can shift dramatically towards an enrichment of antimicrobial resistant populations even after a short exposure to antibiotics. This also highlights the potential of soil to harbour native AMR bacteria, for these microbes to become dominant, and subsequently spread after exposure to antibiotics. The long-term consequence of shifts in community composition, for example biogeochemical transformations, soil fertility, and disease risk is not clear [25].

The resistome in soil could be a result of in situ selection as a consequence of natural production of antimicrobials. Indeed, soils intrinsically harbour AMR bacteria and are a natural reservoir for ARGs [7, 12]. Alternatively, antimicrobial resistant bacteria or ARGs could have been introduced to the soil from external sources. This is common in soils exposed to livestock manure or sludge [9, 18]. Moreover, the dispersal through unconventional sources such as birds can provide the initial seed for the microbial communities to spread AMR. Birds have been shown to spread AMR through long-distance and localised migration [26, 27]. For example, Franklin’s gulls (*Leucophaeus pipixcan*) in Chile were found to have twice the prevalence of ESBL-producing *E. coli* compared to humans in the same area along with high sequence similarity suggesting transmission. The gulls shared sequences with drug-resistant human pathogens identified in clinical isolates from the central Canadian region, which is a nesting place [27]. However, more studies are needed to decisively establish the roles of birds to encounter and acquire active antimicrobial resistant microbes in soils without prior exposure to antibiotic.

The active pool of antimicrobial resistant microbes was dominated by Pseudomonadota, Actinomycetota, Acidobacteriota (Figs. 4 and 5). Pseudomonadota are known to be physiologically and metabolically versatile with variable morphology that allows them to subsist in various ecological niches [28–33]. This could be the reason why 72–84% of the OTUs labelled in the presence of antibiotics were affiliated to Pseudomonadota (Figs. 4 and 5, S1, S2). Further, due to their versatility, Pseudomonadota also contain the greatest number of bacterial pathogens to an extent that this phylum has been proposed to be a potential diagnostic signature for disease risk [34, 35]. Actinomycetota is another near ubiquitous phylum in soil that are known for their ability to synthesize diverse secondary metabolites and harbour different ARGs [33, 36, 37]. It is hypothesised that in soils, ARGs of pathogenic Pseudomonadota originated from Actinomycetota through horizontal gene transfer using conjugative plasmids [38–40]. These results reaffirm the role of Actinomycetota in AMR spread and high abundance in AMR microbiomes. Similarly, Acidobacteriota is also widespread in soil with phylogenetic depth and ecological importance comparable to Pseudomonadota [33, 41]. Acidobacteriota can harbour multiple integrative and conjugative elements in their genome, a major determinant of horizontal gene transfer, that confers them a major advantage to survive, resist, and persist in the presence of antibiotic [42, 43].

In this study, *Stenotrophomonas* was found to be the dominant genus with a relative abundance of 76% in the active resistant microbiome (Figs. 4, 5 and 6) and it possessed ARGs for diverse antibiotics (see MAG-1 in Table 1). *Stenotrophomonas* is an antibiotic resistant opportunistic pathogen that is commonly linked to respiratory infections in humans [44]. Possession of a wide range of ARGs by *Stenotrophomonas* in an antibiotic unexposed soil is disturbing, but not unusual and rare. For instance, on one hand, ARG in *Stenotrophomonas* strains has been reported from deep-sea invertebrates [45]. On the other hand, multi-drug resistant *Stenotrophomonas* is a common nosocomial and community-acquired infection [44].

The active pool of antimicrobial resistant microbiota in this agricultural soil contained ARGs for a wide variety of antibiotics (Table 1). Surprisingly, many of these antibiotics such as aminoglycosides, chloramphenicol, were not part of the experiment in this study. This highlights the potential role of resistant bacteria in AMR spread. We hypothesise that these microbes are present in soils at low abundance but with selection can become enriched increasing the probability of causing disease outbreaks in livestock and human populations. Their enrichment may also spread resistance within the microbial community through HGT.

## Conclusion

In this study, the active resistant soil microbiome from an agricultural field with no prior history of antibiotic exposure using DNA-SIP with H_2_^18^O was identified and differences in the composition of active soil microbes and active antimicrobial resistant soil microbes were observed. The metagenome data shed light on the diversity of antimicrobial resistant genes of the resistant microbiome. We identified the prevalence of antimicrobial resistant *Stenotrophomonas* in the soil, which might be consequential for AMR spread and disease risk. Overall, this study makes a strong case for DNA-SIP with H_2_^18^O to identify the clinically important drug-resistant microbes in the environment. Finally, this method can become gold standard to understand and identify the drivers of AMR spread in any environment.

## Materials and methods

### Soil sampling

Agricultural soils from Chilworth Manor Experimental plots (Southampton, U.K.) were sampled in October 2016. This soil does not have history of manure or antibiotic applications for at least 20 years (M. Cotton, *pers. comm*.). Samples were collected from 10-cm deep in a 10 m triangular pattern. In total, three independent soil samples were transported to the laboratory and stored at 4 °C for further experiments. Physico-chemical analyses were carried out at the Anglian soil analyses company (Lincolnshire, U.K.) and detailed in Table S1. This soil is a sandy/loam with a pH of 6.17 (± 0.006), organic matter content of 7.73% (± 1.43) and dry matter content of 85.99% (± 3.58).

### Soil incubations

Initial tests were performed to determine the concentration of antibiotic necessary to inhibit bacterial growth in soil for up to 12 days. This was necessary due to potential attenuation of the antibiotic by the soil (for methodology see Appendix S1). Since the attenuation of the antibiotic was as fast as two days (Figure S3), a second preliminary experiment was carried out by incubating the soils with several antibiotics to determine the suitable ones to be used for further labelling experiment. Antibiotics were chosen because of their mechanism of action and described in Appendix S1. After performing the preliminary experiments, we decided to incubate the soils for up to four days due to its fast decomposition (Figure S3) and four antibiotics were chosen for further incubations with H_2_^18^O (Table S2). One g of soil was incubated in 1.5 ml of either labelled water (H_2_^18^O) or unlabelled water (H_2_O). Antibiotics [meropenem (mem), cefotaxime (ctx), ciprofloxacin (cip) and trimethoprim (tmp)] at a concentration of 100 µg/ml each were added to the slurry at time 0 and 48 h. Incubations were performed at 200 rpm, in the dark and at room temperature. For both controls and the treatment, sampling was carried out after four days of incubation. The experiments were performed in triplicate (Figure S4).

### DNA extraction

DNA was extracted from the soil at the end of the treatments by using the PowerSoil DNA isolation kit (Qiagen, UK) according to the manufacturer’s recommendation. DNA purity and quantification were determined using a NanoDrop® Spectrophotometer ND-1000 (Thermo Fisher Scientific, USA). All DNA samples were stored at -80 °C for further analysis.

### H_2_^18^O-SIP procedure

A standard DNA-SIP protocol was used to resolve [^18^O]-incorporation based on buoyant density [46]. 1 µg of genomic DNA was loaded into 5.6-ml polyallomer quick-seal centrifuge tubes (Beckman Coulter, USA) containing gradient buffer and cesium chloride (CsCl) [19]. The isopycnic centrifugation of DNA was performed with an initial CsCl buoyant density of 1.725 g mL^-1^ subjected to centrifugation at 177,000 × g for 36–40 h at 20 °C in an Optima XPN-80 ultracentrifuge (Beckman Coulter, USA). At the end of the centrifugation, 18 fractions were separated from each gradient.

### Quantitative PCR

The 16S rRNA gene was quantified in each of the fractions. All qPCR reactions were performed on a StepOne Plus real-time PCR system (Applied Biosystems) and the data were processed using StepOne software v2.3 (Applied Biosystems). For all assays, standards were prepared by PCR of cloned genes. Standards were serially (10^1^–10^7^) diluted and used for the calibration curves in each assay. The assays were based on dual-labelled probes using the primer–probe sets: BAC338F/BAC516P/BAC805R [47]. Each reaction was 20 µL in volume and contained the following mixture: 10 µL of TaqMan fast advanced master mix (1X) (Applied Biosystems), 1.0 µL of primer mix [18 µL BAC338F (0.9 µM), 18 µL BAC805R (0.9 µM), 5 µL BAC516P (0.25 µM) and 59 µL of TE buffer], DNA template (2.0 µL) and 7.0 µL of water. The program used was 95 °C for 5 min, followed by 35 cycles of 95 °C for 30 s and 62 °C for 60 s for annealing, extension and signal acquisition respectively [48]. Efficiencies of 97 to 103% with R^2^ values > 0.98 were obtained.

### High-throughput sequencing

The 16S rRNA genes from SIP gradient fractions was amplified and sequenced by barcoded Illumina MiSeq sequencing. PCR primers 515FB (GTGYCAGCMGCCGCGGTAA) and 806RB (GGACTACNVGGGTWTCTAAT) from the Earth Microbiome project (http://press.igsb.anl.gov/earthmicrobiome/) targeting the V4 region of the 16S rRNA gene were used. Library preparation and sequencing was performed by the Environmental Sequencing Facility of the University of Southampton, UK, following methodologies described by Caporaso et al. [49].

The total metagenomic DNA of the heavy and light fractions from incubations with H_2_^18^O (total of six samples) were sequenced on an Illumina MiSeq at the University of Southampton. The metagenome was analysed on a high-performance computing cluster supported by the Research and Specialist Computing Support Service at the University of East Anglia (Norwich, UK).

### Bioinformatic analyses

For the 16S rRNA-sequencing, quality filtering of the sequences was carried out by using cutadapt [50]. Forward and reverse reads were then merged by using the usearch fastq_mergepairs command [51]. Downstream processing was performed by using UPARSE [51] and UCHIME pipelines [52]. Briefly, sequences shorter than 250 bp were discarded, singletons were retained, and operational taxonomic units (OTUs) were defined at a sequence identity level of 97%.

For the DNA sequences, reads were checked using FastQC version 0.11.8 [53]. Low-quality reads were discarded using BBDuk version 38.68 [54]. Afterwards, reads were merged into scaffolds using de novo assembler metaSPAdes version 3.13.1 [55]. Binning of the assembled scaffolds from both heavy and light fractions was carried out with the metaWRAP version 1.2.1 [56]. Completion and contamination metrics of the extracted bins were estimated using CheckM [57]. The resulting bins were collectively processed to produce consolidated metagenome-assembled genomes (MAGs) using the bin_refinement module in wetaWRAP.

### Statistical analyses and OTU classification

Statistical analyses were performed using the vegan package [58] in R software version 4.1.1. Tests with P < 0.05 were considered to be statistically significant. Shapiro-Wilk normality test was performed for each analysis. ANOVA was performed when abundance data were normally distributed. A non-parametric Kruskal-Wallis one-way analysis of variance was performed when the data were not normally distributed [59]. In parallel, to test the significance of the differences between 2 samples (i.e., between heavy and light fractions), two-tailed independent t-tests were done.

For all OTU-based statistical analyses, the data set was normalized by a Hellinger transformation [60] using the decostand function. For beta-diversity, principal coordinates analysis (PCoA) ordination of Hellinger distances was carried out using the ‘pcoa’ function. Heatmaps were constructed with ‘pheatmap’ package [61] for the OTUs explaining most of the differences between samples. Principal component analysis (PCA) of the Hellinger transformed data was performed using the prcomp function. The OTUs explaining most of the differences between samples were defined as the 20 OTUs contributing the largest absolute loadings in the first and second dimensions of the PCA [59], obtained from the rotation output file. Hierarchical clustering of the distance matrix was carried out with the “ward.D2” method using ‘hclust’ function.

### Taxonomy analysis

A representative sequence of each OTU was aligned against the SILVA 16S rRNA gene database using the naïve Bayesian classifier (bootstrap confidence threshold of 80%) by using the mothur software platform [62].

The taxonomic classification of the MAG was performed as explained previously [63]. Briefly, DNA–DNA hybridization (dDDH) was conducted using the Type Strain Genome Server (TYGS) [64]. Amino-acid comparisons between the MAG retrieved in this study and their closest relative strains (two-way amino acid identity AAI) were calculated using the enveomics collection [65]. Finally, a phylogenomic tree was created using the automated multi-locus species tree (autoMLST) pipeline [66].

### Antimicrobial resistance genes

Since only one MAG was recovered in this study, the unbinned-assembled reads (from heavy and light fractions) were also analysed. Therefore, all reads (MAG-1, unbinned heavy fractions and unbinned light fractions) were screened for antimicrobial resistance genes (ARGs) using the public database Resfinder version 4.1 [67].

## Electronic supplementary material

Below is the link to the electronic supplementary material.

Supplementary Material 1

## Data Availability

Sequence data were deposited in the NCBI Sequence Read Archive (SRA) under accession number PRJNA428598 for 16 S rRNA gene sequences, PRJNA602606 for raw metagenome data, and PRJNA778335 for MAG-1.

## References

[1] Allen HK, Donato J, Wang HH, Cloud-Hansen KA, Davies J, Handelsman J (2010). Call of the wild: antibiotic resistance genes in natural environments. Nat Rev Microbiol.

[2] Popowska M, Rzeczycka M, Miernik A, Krawczyk-Balska A, Walsh F, Duffy B (2012). Influence of soil use on prevalence of tetracycline, streptomycin, and erythromycin resistance and associated resistance genes. Antimicrob Agents Chemother.

[3] Teuber M (2001). Veterinary use and antibiotic resistance. Curr Opin Microbiol.

[4] Willms IM, Kamran A, Aßmann NF, Krone D, Bolz SH, Fiedler F (2019). Discovery of novel antibiotic resistance determinants in forest and grassland soil metagenomes. Front Microbiol.

[5] Zheng Z, Li L, Makhalanyane TP, Xu C, Li K, Xue K (2021). The composition of antibiotic resistance genes is not affected by grazing but is determined by microorganisms in grassland soils. Sci Total Environ.

[6] Hwengwere K, Paramel Nair H, Hughes KA, Peck LS, Clark MS, Walker CA (2022). Antimicrobial resistance in Antarctica: is it still a pristine environment?. Microbiome.

[7] Nesme J, Cécillon S, Delmont TO, Monier JM, Vogel TM, Simonet P (2014). Large-scale metagenomic-based study of antibiotic resistance in the environment. Curr Biology.

[8] Fan H, Wu S, Dong W, Li X, Dong Y, Wang S et al. Characterization of tetracycline-resistant microbiome in soil-plant systems by combination of H_2_^18^O-based DNA-stable isotope probing and metagenomics. J Hazard Mater. 2021;420:126440. 10.1016/j.jhazmat.2021.126440 34280721

[9] Jadeja NB, Worrich A (2022). From gut to mud: dissemination of antimicrobial resistance between animal and agricultural niches. Environ Microbiol.

[10] van Goethem MW, Pierneef R, van de Bezuidt OKI, Cowan DA, Makhalanyane TP. A reservoir of “historical” antibiotic resistance genes in remote pristine Antarctic soils. Microbiome. 2018;6:1–12. 10.1186/s40168-018-0424-5 PMC5824556 29471872

[11] Bi R, Kong Z, Qian H, Jiang F, Kang H, Gu B (2018). High prevalence of * bla * NDM variants among carbapenem-resistant * Escherichia coli * in Northern Jiangsu Province, China. Front Microbiol.

[12] Nesme J, Simonet P (2015). The soil resistome: a critical review on antibiotic resistance origins, ecology and dissemination potential in telluric bacteria. Environ Microbiol.

[13] Chen QL, Cui HL, Su JQ, Penuelas J, Zhu YG. Antibiotic resistomes in plant microbiomes. Trends Plant Sci. 2019;24:530–41. 10.1016/j.tplants.2019.02.010 30890301

[14] Cerqueira F, Christou A, Fatta-Kassinos D, Vila-Costa M, Bayona JM, Piña B (2020). Effects of prescription antibiotics on soil- and root-associated microbiomes and resistomes in an agricultural context. J Hazard Mater.

[15] Urra J, Alkorta I, Mijangos I, Epelde L, Garbisu C (2019). Application of sewage sludge to agricultural soil increases the abundance of antibiotic resistance genes without altering the composition of prokaryotic communities. Sci Total Environ.

[16] Foley JA, Ramankutty N, Brauman KA, Cassidy ES, Gerber JS, Johnston M (2011). Solutions for a cultivated planet. Nature.

[17] Soils for nutrition: state of the art. FAO; 2022. Available online at https://www.fao.org/3/cc0900en/cc0900en.pdf

[18] Thanner S, Drissner D, Walsh F (2016). Antimicrobial resistance in agriculture. mBio.

[19] Dumont MG, Hernández M (2019). Stable isotope probing.

[20] Schwartz E (2007). Characterization of growing microorganisms in soil by stable isotope probing with H218O. Appl Environ Microbiol.

[21] Aanderud ZT, Lennon JT (2011). Validation of heavy-water stable isotope probing for the characterization of rapidly responding soil bacteria. Appl Environ Microbiol.

[22] Spohn M, Pötsch EM, Eichorst SA, Woebken D, Wanek W, Richter A (2016). Soil microbial carbon use efficiency and biomass turnover in a long-term fertilization experiment in a temperate grassland. Soil Biol Biochem.

[23] Blazewicz SJ, Hungate BA, Koch BJ, Nuccio EE, Morrissey E, Brodie EL (2020). Taxon-specific microbial growth and mortality patterns reveal distinct temporal population responses to rewetting in a California grassland soil. ISME J.

[24] Purcell AM, Hayer M, Koch BJ, Mau RL, Blazewicz SJ, Dijkstra P (2022). Decreased growth of wild soil microbes after 15 years of transplant-induced warming in a montane meadow. Glob Chang Biol.

[25] Reed HE, Martiny JBH. Testing the functional significance of microbial composition in natural communities. FEMS Microbiol Ecol. 2007;62:161–70. 10.1111/j.1574-6941.2007.00386.x 17937673

[26] Hernández J, González-Acuña D. Anthropogenic antibiotic resistance genes mobilization to the polar regions. Infect Ecol Epidemiol. 2016;6:32112. 10.3402/iee.v6.32112 PMC5149653 27938628

[27] Hernandez J, Johansson A, Stedt J, Bengtsson S, Porczak A, Granholm S (2013). Characterization and comparison of extended-spectrum β-lactamase (ESBL) resistance genotypes and population structure of *Escherichia coli* isolated from Franklin’s gulls (*Leucophaeus pipixcan*) and humans in Chile. PLoS One.

[28] Degli Esposti M (2014). Bioenergetic evolution in Proteobacteria and mitochondria. Genome Biol.

[29] Dantas G, Sommer MOA, Oluwasegun RD, Church GM. Bacteria subsisting on antibiotics. Science. 2008;320:100–3. 10.1126/science.1155157 18388292

[30] Paun VI, Lavin P, Chifiriuc MC, Purcarea C (2021). First report on antibiotic resistance and antimicrobial activity of bacterial isolates from 13,000-year old cave ice core. Sci Rep.

[31] Bradley PH, Pollard KS (2017). Proteobacteria explain significant functional variability in the human gut microbiome. Microbiome.

[32] Campbell BJ, Engel AS, Porter ML, Takai K (2006). The versatile ε-proteobacteria: key players in sulphidic habitats. Nat Rev Microbiol.

[33] Delgado-Baquerizo M, Oliverio AM, Brewer TE, Benavent-González A, Eldridge DJ, Bardgett RD et al. A global atlas of the dominant bacteria found in soil. Science. 2018;359:320–5. 10.1126/science.aap9516 29348236

[34] Shin NR, Whon TW, Bae JW (2015). Proteobacteria: microbial signature of dysbiosis in gut microbiota. Trends Biotechnol.

[35] Rizzatti G, Lopetuso LR, Gibiino G, Binda C, Gasbarrini A (2017). Proteobacteria: a common factor in human diseases. Biomed Res Int.

[36] D’Costa VM, McGrann KM, Hughes DW, Wright GD. Sampling the antibiotic resistome. Science. 2006;311:374–7. 10.1126/science.1120800 16424339

[37] Fatahi-Bafghi M (2019). Antibiotic resistance genes in the Actinobacteria phylum. Eur J Clin Microbiol Infect Dis.

[38] Benveniste R, Davies J. Aminoglycoside antibiotic-inactivating enzymes in Actinomycetes similar to those present in clinical isolates of antibiotic-resistant bacteria. Proc Natl Acad Sci USA. 1973;70:2276–80. 10.1073/pnas.70.8.2276 PMC433717 4209515

[39] Klümper U, Riber L, Dechesne A, Sannazzarro A, Hansen LH, Sørensen SJ (2014). Broad host range plasmids can invade an unexpectedly diverse fraction of a soil bacterial community. ISME J.

[40] Jiang X, Ellabaan MMH, Charusanti P, Munck C, Blin K, Tong Y (2017). Dissemination of antibiotic resistance genes from antibiotic producers to pathogens. Nat Commun.

[41] Barns SM, Takala SL, Kuske CR. Wide distribution and diversity of members of the bacterial kingdom Acidobacterium in the environment. Appl Environ Microbiol. 1999;65:1731. 10.1128/aem.65.4.1731-1737.1999 PMC91244 10103274

[42] Gonçalves OS, Santana MF (2021). The coexistence of monopartite integrative and conjugative elements in the genomes of Acidobacteria. Gene Elsevier.

[43] Challacombe J, Kuske C (2012). Mobile genetic elements in the bacterial phylum Acidobacteria. Mob Genet Elements.

[44] Brooke JS. *Stenotrophomonas maltophilia*: an emerging global opportunistic pathogen. Clin Microbiol Rev. 2012;25:2–41. 10.1128/CMR.00019-11 PMC3255966 22232370

[45] Romanenko LA, Uchino M, Tanaka N, Frolova GM, Slinkina NN, Mikhailov V (2008). Occurrence and antagonistic potential of *Stenotrophomonas* strains isolated from deep-sea invertebrates. Arch Microbiol.

[46] Neufeld JD, Vohra J, Dumont MG, Lueders T, Manefield M, Friedrich MW et al. DNA stable-isotope probing. Nature Protocols. 2007;2:860–6. 10.1038/nprot.2007.109 17446886

[47] Yu Y, Lee C, Kim J, Hwang S. Group-specific primer and probe sets to detect methanogenic communities using quantitative real-time polymerase chain reaction. Biotechnol Bioeng. 2005;89:670–9. 10.1002/bit.20347 15696537

[48] Hernández M, Calabi M, Conrad R, Dumont MG (2020). Analysis of the microbial communities in soils of different ages following volcanic eruptions. Pedosphere.

[49] Caporaso JG, Lauber CL, Walters WA, Berg-Lyons D, Huntley J, Fierer N (2012). Ultra-high-throughput microbial community analysis on the Illumina HiSeq and MiSeq platforms. ISME J.

[50] Martin M (2011). Cutadapt removes adapter sequences from high-throughput sequencing reads. EMBnet J.

[51] Edgar RC (2013). UPARSE: highly accurate OTU sequences from microbial amplicon reads. Nat Methods.

[52] Edgar RC, Haas BJ, Clemente JC, Quince C, Knight R (2011). UCHIME improves sensitivity and speed of chimera detection.

[53] Andrews S. FastQC: a quality control tool for high throughput sequence data. 2018. Available online at https://www.bioinformatics.babraham.ac.uk/projects/fastqc/.

[54] Bushnell B, Rood J, Singer E (2017). BBMerge – accurate paired shotgun read merging via overlap. PLoS One.

[55] Nurk S, Meleshko D, Korobeynikov A, Pevzner PA (2017). metaSPAdes: a new versatile metagenomic assembler. Genome Res.

[56] Uritskiy G, Diruggiero J, Taylor J. MetaWRAP - a flexible pipeline for genome-resolved metagenomic data analysis. Microbiome. 2018;6:1–13. 10.1186/s40168-018-0541-1 PMC6138922 30219103

[57] Parks DH, Imelfort M, Skennerton CT, Hugenholtz P, Tyson GW (2015). CheckM: assessing the quality of microbial genomes recovered from isolates, single cells, and metagenomes. Genome Res.

[58] Oksanen J, Blanchet FG, Friendly M, Kindt R, Legendre P, Mcglinn D et al. Vegan: community ecology package. R package version 2.5-2. Available online at https://cran.r-project.org/web/packages/vegan/index.html

[59] Hernández M, Conrad R, Klose M, Ma K, Lu Y (2017). Structure and function of methanogenic microbial communities in soils from flooded rice and upland soybean fields from Sanjiang plain, NE China. Soil Biol Biochem.

[60] Legendre P, Gallagher ED (2001). Ecologically meaningful transformations for ordination of species data. Oecologia.

[61] Kolde R. Package ‘pheatmap’. R package version 1.0.12. 2019. https://cran.r-project.org/web/packages/pheatmap/index.html

[62] Schloss PD, Westcott SL, Ryabin T, Hall JR, Hartmann M, Hollister EB (2009). Introducing mothur: Open-source, platform-independent, community-supported software for describing and comparing microbial communities. Appl Environ Microbiol.

[63] Islam T, Hernández M, Gessesse A, Murrell JC, Øvreås L (2021). A novel moderately thermophilic facultative methylotroph within the class alphaproteobacteria. Microorganisms.

[64] Meier-Kolthoff JP, Göker M (2019). TYGS is an automated high-throughput platform for state-of-the-art genome-based taxonomy. Nat Commun.

[65] Rodriguez -RLM, Konstantinidis KT. The enveomics collection: a toolbox for specialized analyses of microbial genomes and metagenomes. PeerJ Preprints. 2016;4:e1900v1.

[66] Alanjary M, Steinke K, Ziemert N (2019). AutoMLST: an automated web server for generating multi-locus species trees highlighting natural product potential. Nucleic Acids Res.

[67] Florensa AF, Kaas RS, Clausen PTLC, Aytan-Aktug D, Aarestrup FM (2022). ResFinder – an open online resource for identification of antimicrobial resistance genes in next-generation sequencing data and prediction of phenotypes from genotypes. Microb Genom.

