# Supplementary material for: Identification of diverse antibiotic resistant bacteria in agricultural soil with H218O stable isotope probing combined with high-throughput sequencing
Source: Environ Microbiome. 2023 Apr 18;18:34. doi: 10.1186/s40793-023-00489-7 (PMC10111737; doi:10.1186/s40793-023-00489-7)
Supplement: Supplementary file 1 — Supplementary Material 1 [file 40793_2023_489_MOESM1_ESM.pdf]

Supplementary information for

Identification of diverse antibiotic resistant bacteria in agricultural soil with $H_2^{18}O$ stable isotope probing combined with high-throughput sequencing

Marcela Hernández[a,b], Shamik Roy[b], C. William Keevil[a], Marc G. Dumont[a*]

[a] School of Biological Sciences, University of Southampton, Southampton SO17 1BJ, UK

[b] School of Biological Sciences, University of East Anglia, Norwich NR4 7TJ, UK

Running title: Multi-drug resistant bacteria in agricultural soil

*Corresponding author:

Marc G. Dumont, University of Southampton, Life Sciences Building 85, Highfield Campus, Southampton SO17 1BJ, UK, m.g.dumont@soton.ac.uk

Appendix S1: Supplementary Methods

Preliminary experiment 1: Incubation procedures

Meropenem (Sigma Aldrich, UK) at a concentration of 50 µg/ml was added to the soil (2 g) and incubated in water (10 ml) for 12 days at 180 rpm, room temperature (~22°C) and dark. Meropenem was added to the slurry incubations every three days. Samples (15 µl) were taken every day and also before and after every addition of the antibiotic. Agar diffusion test were performed by using LB Agar (tryptone 10 g/l, yeast extract 5 g/l, NaCl 5 g/l, Agar 15 g/l) media and *Escherichia coli* K12. Plates were incubated at 37°C and the presence of halo were measured after incubation.

Preliminary experiment 2: Incubation procedures for multi-drug test

An incubation with multi-drug test was performed in order to determine the suitable antibiotics to be used for further labelling experiment. Antibiotics were chosen because of their mechanism action, i.e, cell was synthesis action: cefotaxime (ctx) and meropenem (mem); protein synthesis action: gentamicin (gen) and amikacin (ami) for their 30S subunit synthesis inhibition and erythromycin (ery) for its 50S subunit synthesis inhibition; nucleid acid synthesis action: ciprofloxacin (cip) for its DNA gyrase synthesis inhibition; trimethoprim (tmp), sulfamethizole (smz) for their folate synthesis inhibition; rifampicin (rif) for its RNA polymerase synthesis inhibition. These antibiotics have been found in the genome of *Klebsiella pneumonia* [1]. Antibiotics (50 µg/ml) was added to soil (1 g) and incubated with 1 ml of water at 180 rpm, dark and room temperature for 4 days. Antibiotics were added at the beginning of the incubation and after 48 h.

References

1. Doi Y, Hazen TH, Boitano M, Tsai YC, Clark TA, Korlach J, et al. Whole-genome assembly of Klebsiella pneumoniae coproducing NDM-1 and OXA-232 carbapenemases using single-molecule, real-time sequencing. Antimicrob Agents Chemother. American Society for Microbiology; 2014;58:5947–53.

Table S1. Physico-chemical parameters from Chilworth agricultural soils

	Soil 1	Soil 2	Soil 3
pH	6.2	6.1	6.2
P (mg/l)	107	117	126
K (mg/l)	219	175	216
Mg (mg/l)	89	150	110
Total N (g/kg)	5.25	4.2	5.6
dry matter %	89.88	82.85	85.23
Cu (mg/l)	9.15	9.15	10.7
Org. matter (%)	6.5	9.3	7.4

Table S2: Heat map illustrating growth in all replicates (dark grey), in some of the replicates (light grey) and no growth of selected antibiotics and their combination with meropenem (mem) during a 4-day incubation of soil with water. ctx: cefotaxime, mem: meropenem; gen: gentamicin; ami: amikacin; ery: erythromycin; cip: ciprofloxacin; tmp: trimethoprim; smz: sulfamethizole; rif: rifampicin.

Antibiotic	d-1	d-2	d-2*	d-3	d-4	Antibiotic	d-1	d-2	d-2*	d-3	d-4
ctx						ctx+mem					
gen						gen+mem					
ami						ami+mem					
cip						cip+mem					
tmp						tmp+mem					
smz						smz+mem					
rif						rif+mem					
ery						ery+mem					
mem						all together					

Antibiotic still present in the slurry for all 3 replicates
antibiotic still present in the slurry for at least 1 replicate
antibiotic not present in the slurry
*second addition of the antibiotic

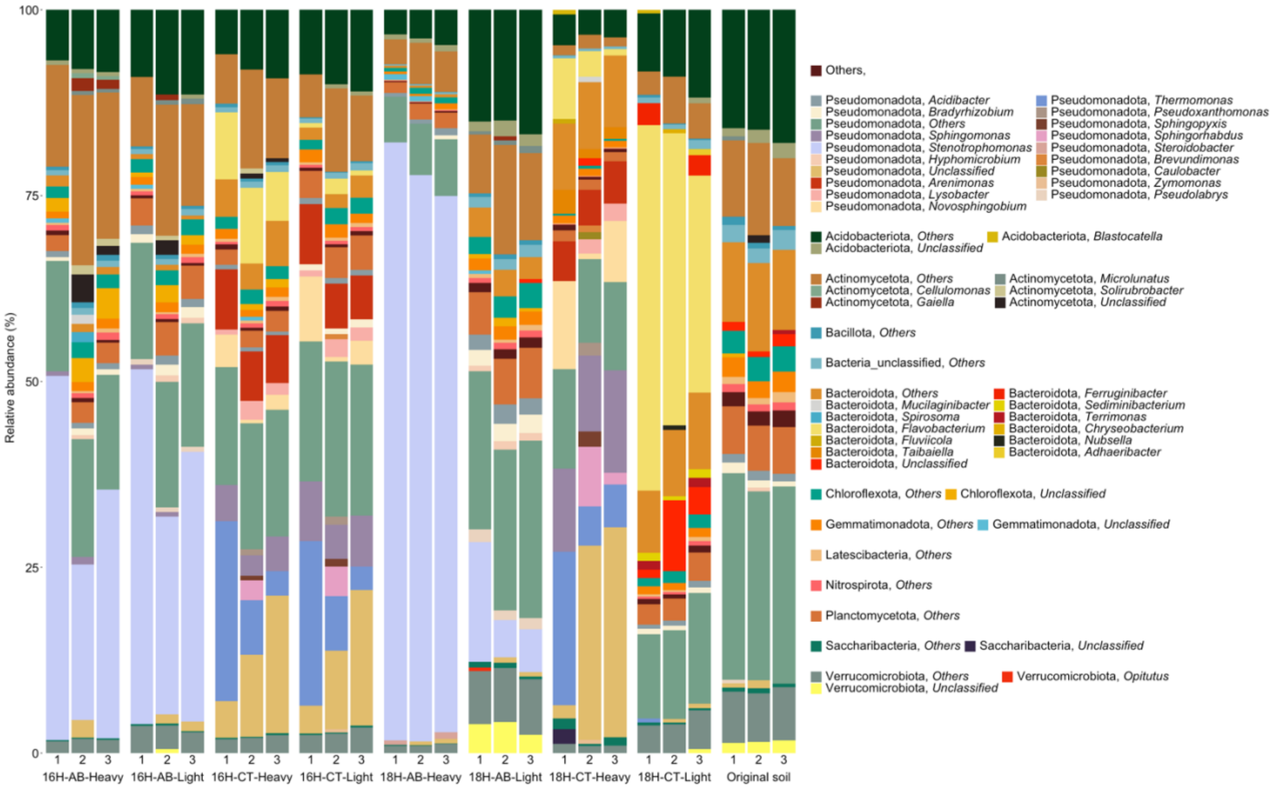

Figure S1. Relative abundance of microbial communities at the phylum level identified in the "heavy" and "light" fractions of DNA extracted from soils incubated with $H_2{}^{18}O$ and $H_2{}^{16}O$ in the presence and absence of antibiotics. 16H: incubation with $H_2{}^{16}O$; 18H: incubation with $H_2{}^{18}O$; AB: incubation with antibiotics; CT: incubation without antibiotics; Heavy: "heavy" fractions of the extracted soil DNA; Light: "light" fractions of the extracted soil DNA.

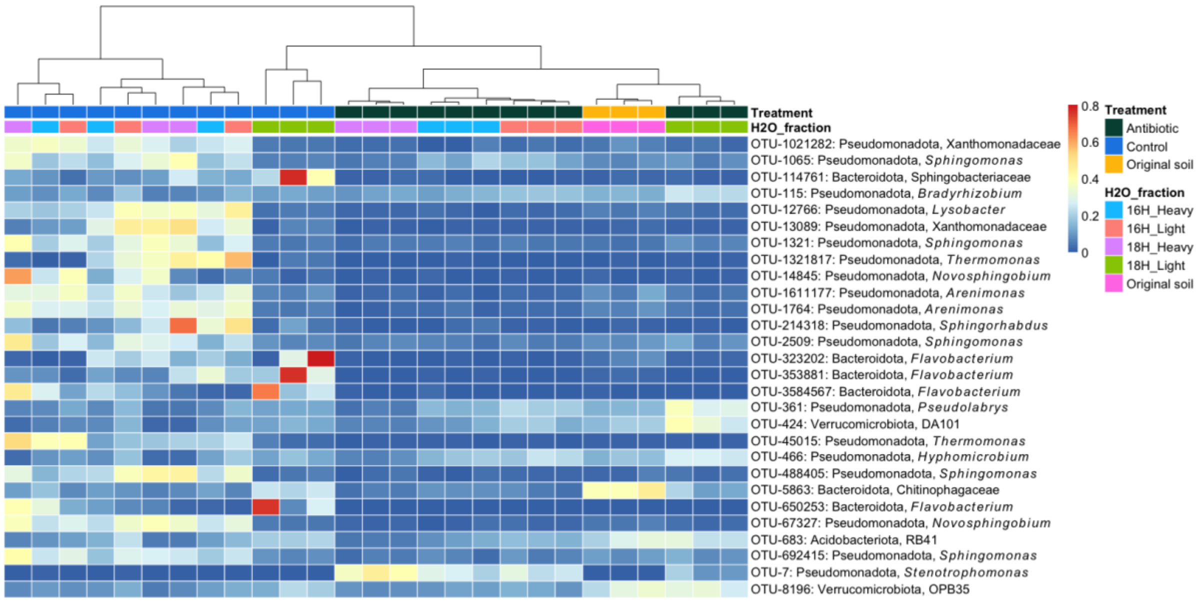

Figure S2. Heatmap of the most relevant bacterial OTUs identified in the "heavy" and "light" fractions of DNA extracted from soils incubated with $H_2{}^{18}O$ and $H_2{}^{16}O$ in the presence and absence of antibiotics. 16H: incubation with $H_2{}^{16}O$; 18H: incubation with $H_2{}^{18}O$; AB: incubation with antibiotics; CT: incubation without antibiotics; Heavy: "heavy" fractions of the extracted soil DNA; Light: "light" fractions of the extracted soil DNA.

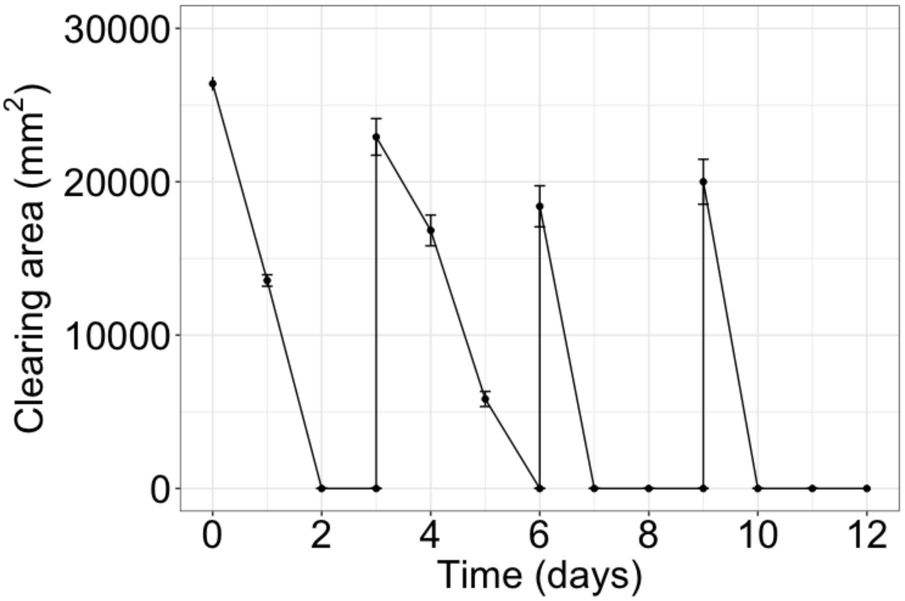

Figure S3. Degradation kinetics of meropenem during 12 days of incubation. Meropenem was added to the slurry every 2 days. Values are average and bars above the values indicate standard deviation of triplicates.

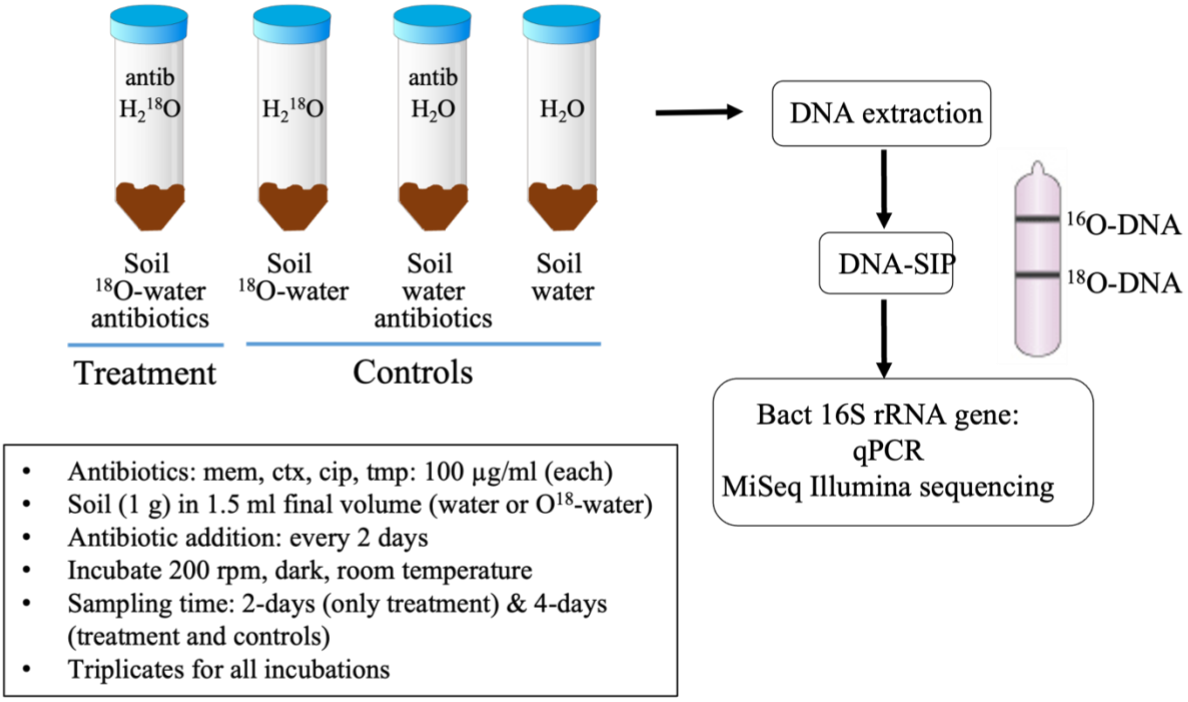

Figure S4. Scheme depicting the setting-up of the labelling incubations with antibiotics and $H_2{}^{18}O$.
